# Oral microbiome-driven virulence factors: A novel approach to pancreatic cancer diagnosis

**DOI:** 10.17305/bb.2023.9934

**Published:** 2024-08-01

**Authors:** Xuemin Zeng, Dapeng Ren, Ran Liu, Qiang Zhang, Xiao Yan, Xiao Yuan

**Affiliations:** 1 Department of Stomatology Medical Center, Affiliated Hospital of Qingdao University, Qingdao University, Qingdao, China; 2 Central Laboratory of Affiliated Hospital of Qingdao University, Qingdao University, Qingdao, China; 3 Department of Orthodontics, School of Stomatology, Qingdao University, Qingdao, China

**Keywords:** Pancreatic cancer, oral microbiome, virulence factors (VFs), non-invasive diagnostic, saliva

## Abstract

Pancreatic ductal adenocarcinoma (PDAC) is a highly aggressive malignancy, often associated with a poor prognosis for patients. One of the major challenges in managing PDAC is the difficulty in early diagnosis, owing to the limited and invasive nature of current diagnostic methods. Recent studies have identified the oral microbiome as a potential source of noninvasive biomarkers for diseases, including PDAC. In this study, we focused on leveraging the differential expression of virulence factors (VFs) encoded by the oral microbiome to create a diagnostic tool for PDAC. We observed a higher alpha diversity in VF categories among PDAC patients compared to healthy controls. We then identified a panel of VF categories that were significantly upregulated in PDAC patients, these being associated with bacterial adherence, exoenzyme production, and nutritional/metabolic processes. Moreover, *Streptococcus*-derived VFs were notably enriched in PDAC patients. We developed a diagnostic model using random forest analysis based on the levels of these VFs. The model’s diagnostic accuracy was evaluated using receiver operating characteristic (ROC) curve analysis, with an area under the curve (AUC) of 0.88, indicating high accuracy in differentiating PDAC patients from healthy controls. Our findings suggest that VFs encoded by the oral microbiome hold potential as diagnostic tools for PDAC, offering a non-invasive approach that could significantly enhance early detection and prognosis, ultimately leading to improved patient outcomes.

## Introduction

Pancreatic ductal adenocarcinoma (PDAC) ranks among the most lethal cancers, characterized by its aggressive nature and poor patient prognosis [1–3]. The early detection of PDAC remains a significant challenge due to the limited and invasive nature of current diagnostic methods, with early detection being critical for improving patient outcomes [2, 3]. The gut microbiota, consisting of trillions of microorganisms inhabiting the human intestines, and their composition and function have been closely linked to overall health and disease development [4–6]. In recent years, emerging evidence suggests the potential role of gut microbiota in the diagnosis and prognosis of various diseases, including PDAC [1, 7, 8].

Additionally, the oral microbiome, comprising a complex ecosystem of bacteria, viruses, fungi, and other microorganisms, is crucial for oral health and disease [4]. Emerging evidence suggests that the oral microbiome plays a crucial role in the pathogenesis of several diseases, including oral and gastrointestinal cancers [9]. Understanding the interaction between the oral microbiome and PDAC is paramount for identifying potential diagnostic and therapeutic strategies. Recent research has shed light on the potential of the oral microbiome, the diverse community of microorganisms residing in the oral cavity, as a non-invasive source of biomarkers for various diseases, including PDAC [4]. However, current diagnostic accuracy using oral or gut microbiota is about 80% (0.78–0.82), indicating a need for improvement [4].

In recent years, the focus has shifted toward understanding the specific mechanisms by which these microorganisms can induce pathogenic effects [10, 11]. One such mechanism relies on the presence of virulence factors (VFs) encoded by the microbial genome [12]. VFs are molecular components or proteins produced by microorganisms that enhance their ability to colonize and infect host tissues. However, utilizing VFs to reflect the microbiome’s virulence characteristics for disease diagnosis [13] poses feasibility challenges.

This study aims to investigate the association between PDAC and VFs encoded by the oral microbiome, representing the first characterization of VF features in the oral microbiome of PDAC patients. Our study demonstrates the potential of utilizing the VFs encoded by the oral microbiome as diagnostic biomarkers for PDAC.

## Materials and methods

### Data collection

**Figure 1.**
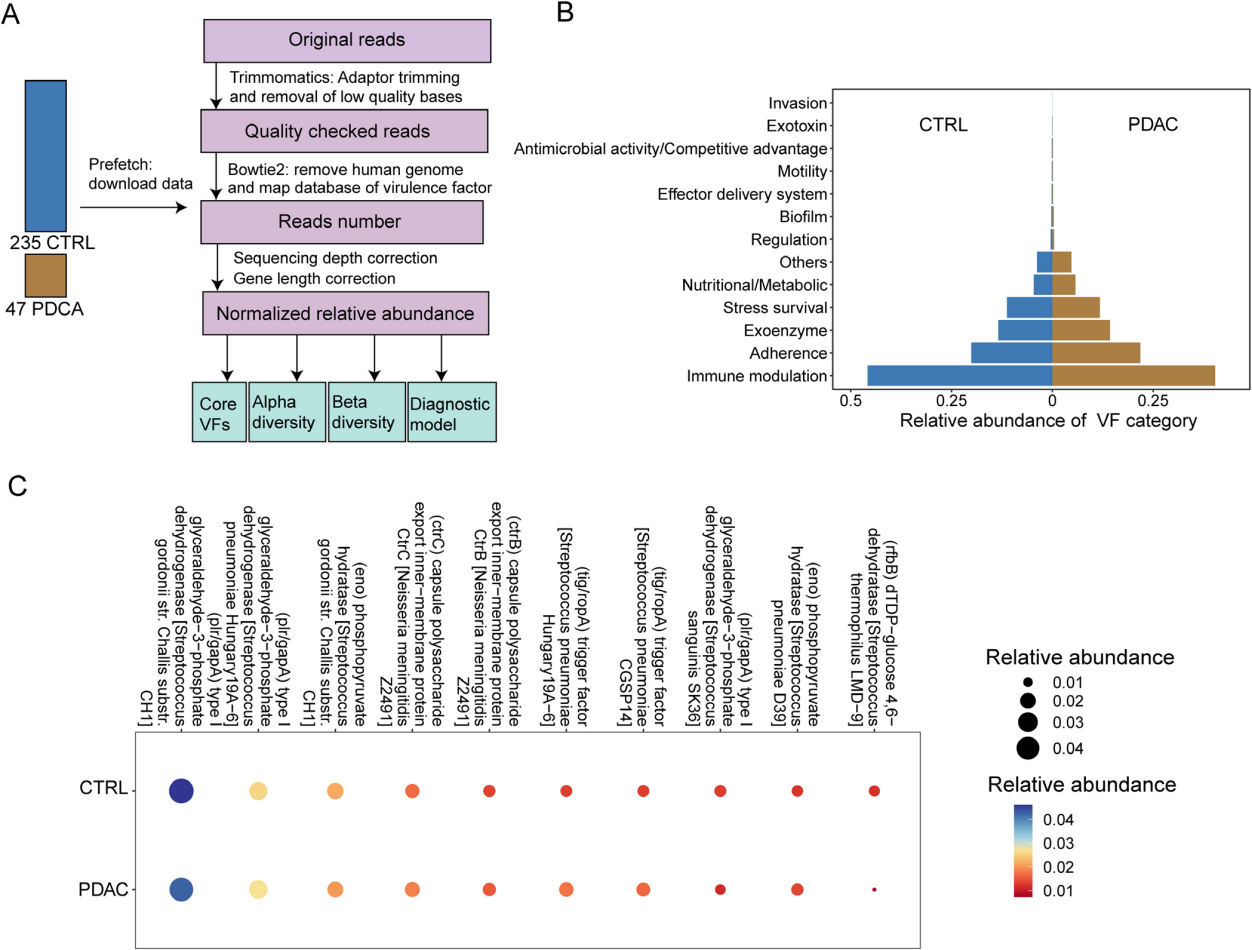
**The analysis workflow and core VF categories and individual VFs.** (A) Data acquisition and processing workflow; (B) Relative abundance of VF category; (C) Top ten relative abundance of VF across all samples. CTRL: Control; PDAC: Pancreatic ductal adenocarcinoma; VF: Virulence factor.

Nagata et al. [1] conducted a study in which 47 salivary samples were collected from patients diagnosed with PDAC, while 235 samples were obtained from healthy individuals as controls. The authors considered potential confounding factors like gender and age in both groups. Importantly, based on their original *P* value, these confounding factors had limited impact within the dataset we used [1]. Therefore, in our study, we did not account for these factors due to their minimal influence on our analysis. For data retrieval, we utilized the prefetch tool v2.10.7 from the National Center for Biotechnology Information (NCBI), allowing us to download the required datasets. Our data collection and processing steps are outlined in Figure 1A, providing a visual representation of the entire workflow.

### Quality control of shotgun metagenomic sequence

To ensure sequencing data quality, we used Trimmomatic v0.39 [14] for the removal of adapter sequences and low-quality bases (using the following parameters, ILLUMINACLIP: TruSeq3-PE-2.fa:2:30:10:8:true TRAILING:20 MINLEN:60, to trim the ends of reads with a quality below 20; and MINLEN:60, to discard any processed reads shorter than 60 base pairs). This step involved precision trimming of adapter sequences, a known source of potential contamination in sequencing data, as well as the excision of low-quality bases that could compromise downstream analysis. After quality control, the reads underwent an additional processing step to remove human genomic sequences using bowtie v2.4.4 [15] and the T2T-mY-rCRS genome [16] (with hard-masked pseudoautosomal regions [PARs] on chrY replaced with “N” and mitochondrion replaced with revised Cambridge reference sequence [rCRS]), available at https://github.com/marbl/CHM13. This approach aimed to effectively remove any human genomic contamination, particularly in saliva samples characterized by a high host genomic content.

### Virulence factor (VF) annotation

After eliminating human genomic sequences, we proceeded to align the remaining reads against the VF database (VFDB) [10]. This database, available at http://www.mgc.ac.cn/VFs/main.htm, provides a comprehensive collection of VFs. For the alignment process, we employed bowtie v2.4.4 [15] and utilized samtools v1.13 [17] for subsequent analysis. The alignment tool (bowtie2) is optimal as it handles large datasets efficiently, ensuring that even the subtlest of microbial sequences can be matched to known VFs. By aligning the reads against the VFDB_setB_nt (http://www.mgc.ac.cn/VFs/download.htm), we were able to identify the number of reads that corresponded to VFs in each sample. To ensure accurate quantification of their abundance, we implemented standardization measures: one that accounts for the depth of sequencing in each sample—a measure of the extent to which the microbial content has been sampled—and another that adjusts for the length of the VF genes identified. This dual-normalization process is critical, as it compensates for both technical and biological variance, allowing the comparative abundance of VFs across samples to accurately reflect their genuine prevalence [18]. The standardized relative abundance will be utilized in subsequent analyses, providing a comprehensive understanding of the oral microbiome-encoded VFs in PDAC.

### Diagnostic model construction

We developed a random forest [19] classifier based on VFs related to the oral microbiome for PDAC diagnosis. Our dataset comprised genomic features associated with VFs, with the aim of leveraging these features for accurate pancreatic cancer diagnosis. Initially, we loaded the dataset, which contained information on various genomic features and their corresponding disease outcomes. To perform the analysis, we divided the dataset into input features (*X*) and target variables (*y*), where *X* represented the genomic features and *y* represented the disease outcomes. To ensure the selection of the most relevant genomic features, we performed a correlation analysis. This involved calculating Pearson correlation coefficients between each feature and the target variable. The dataset was split into a training set and a test set, using 80% of the data for training the model and reserving 20% for evaluating its performance. Subsequently, we constructed a random forest classifier, optimizing parameters, such as the number of decision trees and their maximum depth, to enhance model performance. The classifier consisted of the trees to optimize the model’s performance on the test set. We have determined through model validation that a random forest comprising 100 trees strikes a reasonable balance between predictive performance and computational efficiency, and the maximum depth of trees was no limit on tree depth. We evaluated the model’s effectiveness using receiver operating characteristic (ROC) curves and calculated the area under the curve (AUC) values. These metrics provide a quantitative measure of the model’s classification performance.

### Statistical analysis

Statistical analyses were conducted using RStudio. Alpha diversity measures, including Shannon and Simpson indices, were calculated using the vegan package. The Bray–Curtis dissimilarity index was computed directly on VF profiles. Principal coordinate analysis (PCoA) was performed using ade4 [20], and adonis analysis (vegan package) assessed group differences’ significance. Differential VF abundances were tested using the Wilcoxon rank-sum test, with *P* values adjusted by the Benjamini–Hochberg (BH) procedure, with a significance threshold set at a *P* adjust value of <0.05. The ggplot2 package was used for creating boxplots and PCoA plots. The pheatmap package was utilized to construct heatmaps, visualizing the patterns of VF abundances. ROC curves were generated using the pROC package and were utilized to evaluate the performance of diagnostic models.

## Results

### Core VF categories and individual VFs in oral microbiome of PDAC patients

Our analysis workflow, as illustrated in Figure 1A, aimed to determine the sequence abundance of VFs in all samples. We categorized the identified VFs into 13 different categories, and their relative abundances are presented in Figure 1B. Our results revealed that the dominant VFs in the oral microbiome of PDAC patients are primarily categorized under immune modulation (average abundance in controls and PDAC patients: 45.84% and 40.38%, respectively), adherence (20.07%, 21.93%), exoenzyme (13.39%, 14.30%), stress survival (11.24%, 11.77%), and nutritional/metabolic (4.58%, 5.75%). These categories are followed by others (3.78%, 4.74%), regulation (0.41%, 0.44%), biofilm (0.23%, 0.34%), effector delivery system (0.17%, 0.15%), motility (0.13%, 0.13%), antimicrobial activity/competitive advantage (0.11%, 0.11%), exotoxin (<0.1%, <0.1%), and invasion (<0.1%, <0.1%). We also identified the top ten VFs with the highest average relative abundance, predominantly associated with *Streptococcus*, correlating with their increased abundance in the oral cavity of PDAC patients.

### PDAC patients have higher alpha diversity of VF categories

**Figure 2.**
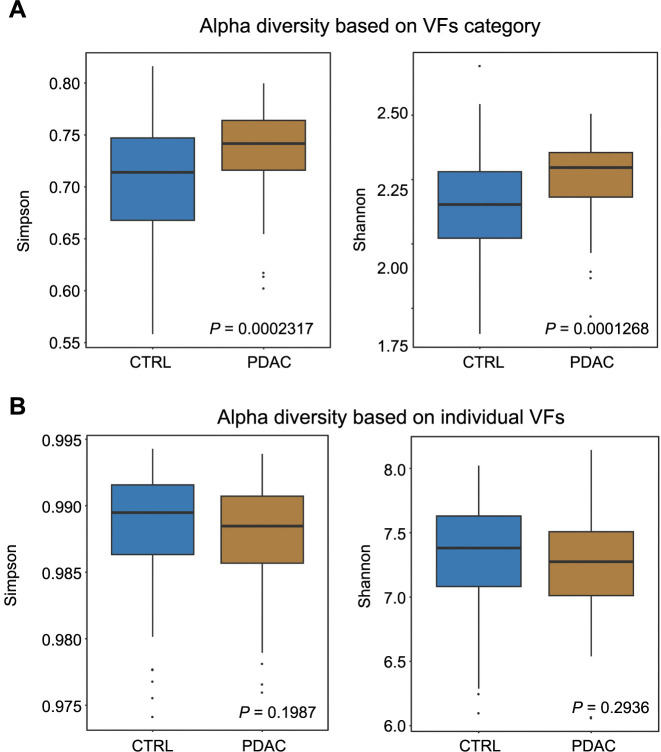
**Alpha diversity based on (A) VF category and (B) individual VF**. Simpson and Shannon indices were calculated. The central line within each box represents the median value, while the top and bottom edges of the box depict the IQR, being the 75th and 25th percentiles, respectively. The points that fall outside of this range represent the outliers within the dataset. CTRL: Control; PDAC: Pancreatic ductal adenocarcinoma; VF: Virulence factor; IQR: Interquartile range.

The alpha diversity, calculated using both Shannon and Simpson indices, based on both VF categories and VFs, found that PDAC patients exhibited higher alpha diversity when considering the 13 VF categories. We found that for the Simpson index, the *P* value was 0.0002317 and a *P* value of 0.0001268 for the Shannon index (Figure 2A). This trend was not observed in the analysis of individual VFs (Simpson: *P* ═ 0.1987, Shannon: *P* ═ 0.2936, Figure 2B). Overall, our findings suggest that PDAC patients’ oral microbiome encodes a broader array of VFs, potentially contributing to oral environment deterioration.

### Differential abundance of VF categories and individual VFs between PDAC and healthy controls

**Figure 3.**
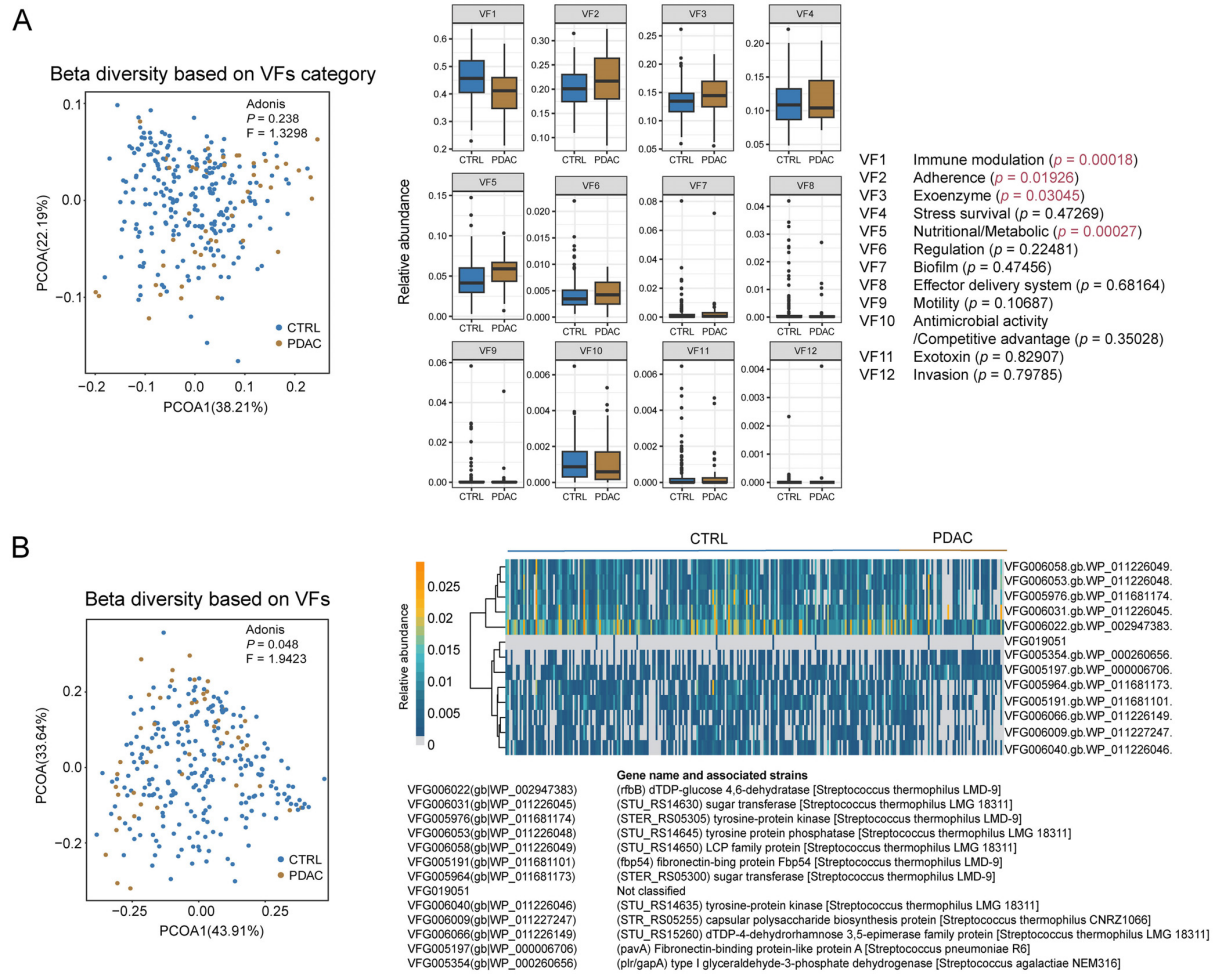
**Beta diversity based on Bray–Curtis dissimilarity index and differential (A) VF category and (B) individual VF.** The *P* values were adjusted using the BH procedure, with a significance threshold set at a *P* adjust value of <0.05. CTRL: Control; PDAC: Pancreatic ductal adenocarcinoma; VF: Virulence factor; BH: Benjamini–Hochberg; PCOA: Principal coordinate analysis.

Subsequently, we calculated beta diversity based on both VF categories and individual VFs. When considering the 13 VF categories, we did not observe significant differences in VF category composition between PDAC patients and healthy controls (*P* ═ 0.238, *F* ═ 1.3298, Figure 3A). We further investigated if there were differences in VF categories. We identified four differentially enriched VF categories, with three being enriched in PDAC (adherence, exoenzyme, and nutritional/metabolic processes) and one being enriched in healthy controls (immune modulation). However, we observed differences in the composition of VFs (*P* ═ 0.048, *F* ═ 1.9423, Figure 3B). We further identified differentially expressed VFs. As expected, we found 13 significantly different VFs (*P* adjust < 0.05) associated with *Streptococcus*. Among them, two VFs were upregulated in PDAC, while 11 VFs were enriched in healthy controls. The above results once again highlight the characteristics of the VF of oral microbiome in PDAC patients.

### VFs for PDAC diagnosis

**Figure 4.**
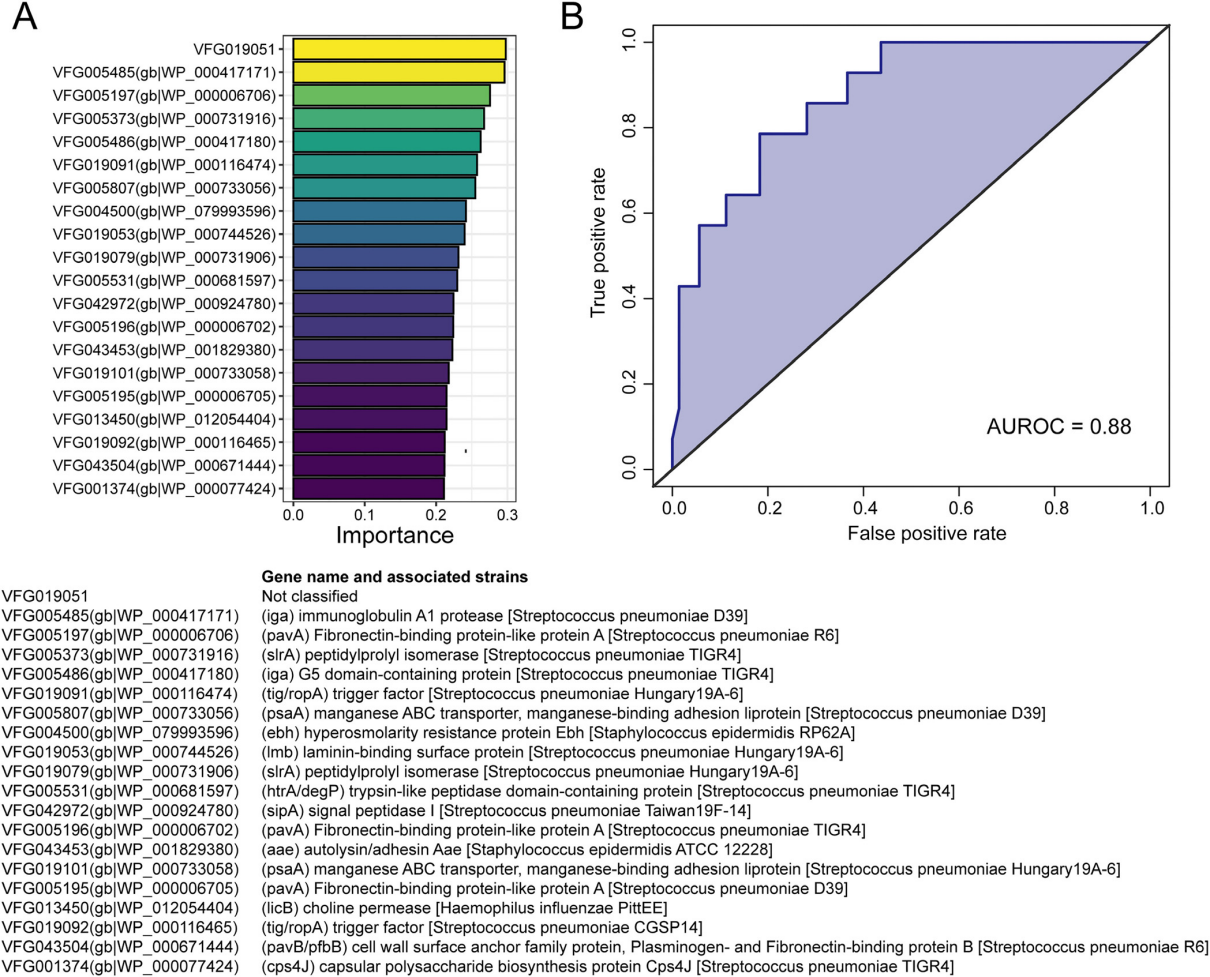
**Construction of the diagnostic model.** (A) Identification of top 20 VFs contributing the most to the model; (B) Evaluation of the accuracy of the diagnostic model. AUROC: Area under the receiver operating characteristic curve; VF: Virulence factor.

The distinctive distribution of VFs in PDAC indicates their potential as a new approach for diagnosing PDAC. To achieve this, we developed a diagnostic model for PDAC using VF profiles, employing a random forest model. In Figure 4A, we showed the top 20 VFs in the model, including the prominent ones like immunoglobulin A1 protease (iga), fibronectin-binding protein-like protein A (pavA), peptidylprolyl isomerase (slrA), G5 domain-containing protein (iga), and trigger factor (tig/ropA). Our results demonstrated high accuracy (AUROC ═ 0.88, Figure 4B) in diagnosing pancreatic cancer, indicating that oral microbiota-encoded VFs could serve as alternative biomarkers.

## Discussion

Attention is now being directed to comprehending the precise processes through which these microbes can cause disease. A key method involves the production of VFs. The identification and development of novel approaches for the early diagnosis of PDAC are of most importance due to the aggressive nature and poor prognosis of this disease [2]. In recent years, increasing evidence suggests that the oral microbiome and its associated VFs play a significant role in disease [21–23]. Most studies have focused on the composition of the oral microbiome at the level of species or strain, aiming to explain the differences between disease and healthy cohorts at this taxonomic level. However, limited research has been conducted on specific gene categories, such as antibiotic resistance genes and VF genes, which could provide novel insights. VFs are molecular properties, typically, gene products, that empower microorganisms to colonize and infect specific host species, thereby increasing their ability to cause disease. These factors encompass a range of characteristics, including bacterial toxins, cell surface proteins that facilitate bacterial attachment, protective cell surface carbohydrates, and proteins, as well as hydrolytic enzymes that contribute to the bacterium’s pathogenicity [10]. To characterize the VF features of the oral microbiome in PDAC patients, we conducted this study. First, we observed that the box-plot graphics lack crucial descriptions for proper interpretation. For instance, clarification is needed regarding the symbolism of the lines and dots. We also observed a noticeable increase in alpha diversity, specifically based on VF categories, in PDAC patients compared to healthy controls. Furthermore, we identified a set of VF categories that exhibited significant upregulation in PDAC patients. These categories were primarily associated with bacterial adherence, exoenzyme production, and nutritional/metabolic processes.

Importantly, our work evaluates the potential of oral microbiome-driven VFs as a promising avenue for pancreatic cancer diagnosis. This is particularly crucial given the typically asymptomatic nature of early-stage PDAC and the demand for noninvasive and cost-effective diagnostic tools. In a previous study, the diagnostic accuracy of using the composition of gut microbiota and oral microbiota for diagnosing PDAC ranged from 0.78 to 0.82 [1]. However, in our study, our model achieved a higher accuracy of 0.88, surpassing the accuracy of the previous models. We hypothesize that the inclusion of these VFs provides a more dynamic and functionally relevant picture of the microbiome’s role in the pathology. By integrating data on VFs, the model is better equipped to infer the potential pathogenic mechanisms and their direct implications for disease severity and patient outcomes. It is these attributes that may offer a more precise and clinically relevant predictive power. 

## Conclusion

Collectively, our study provides an initial comprehensive analysis of VF features within the oral microbiome of PDAC patients. In the absence of such external validation, we still believe that our findings yield valuable insights for the studied population and make a meaningful contribution to the existing body of knowledge. We encourage other research groups to apply our model to different datasets to further investigate its broader applicability and robustness. Additionally, our research demonstrates the promising potential of utilizing VF encoded by the oral microbiome as diagnostic biomarkers for PDAC. It is important that VFs can also serve to monitor disease progression and assess treatment response, like microorganisms. The possibility of altering VFs as a therapeutic strategy alongside traditional PDAC treatment is acknowledged, aligning with research suggesting that certain bacterial populations may contribute to disease pathogenesis. The integration of microbiome analysis into current clinical workflows, considering the technical challenges and the required standardization before such tests can be widely adopted in clinical practice. We believe that further investigation of the specific functions of these VFs or their relationship to the long-term prognosis of PDAC is needed.

## Data Availability

All shotgun metagenomic sequences were collected by Nagata et al. study and can be downloaded from the National Center for Biotechnology Information (NCBI, PRJNA832909) database.
